# A proteomic predictor of conversion from mild cognitive impairment to dementia with potential utility in enhancing productivity of emerging clinical trials

**DOI:** 10.1002/alz.095359

**Published:** 2025-01-09

**Authors:** Clare Paterson, Bryan Dawkins, Hannah Biegel, Yolanda Hagar, Stephen A Williams

**Affiliations:** ^1^ Standard BioTools, Boulder, CO USA; ^2^ SomaLogic, Boulder, CO USA

## Abstract

**Background:**

A significant proportion of individuals with mild cognitive impairment (MCI) develop dementia, with annual conversion rates exceeding 10%. Earlier dementia diagnosis and intervention can improve outcomes, and new disease‐modifying drugs are being repositioned for the preclinical stages of illness. Identifying individuals at risk for conversion from MCI to dementia could offer substantial benefits to public healthcare and pharmaceutical industries. Given the absence of a surrogate endpoint for dementia actual clinical diagnoses are needed to develop in trials. Therefore, we sought to develop a protein‐based tool with potential for trial population enrichment through risk prediction of dementia in individuals with MCI.

**Method:**

Using modified‐aptamer proteomics technology, SomaScan® Assay, we assessed ∼5,000 proteins in 954 plasma samples from individuals aged 67‐89 with diagnosed MCI at visit 5 of the Atherosclerosis Risk in Communities study. In total, 182 (19.1%) converted to all‐cause dementia within 5 years of blood‐draw. Time‐to‐dementia diagnosis was modeled using machine learning methods in 70% of samples as a training dataset, and performance assessed in a 15% holdout sample. The proteomic model was compared to the leading conversion risk factors, including ApoE genotype and age

**Result:**

A 10‐feature protein‐only AFT Weibull model successfully predicted the probability of dementia diagnosis within 5 years of blood draw. Model performance was measured using AUC, which was equal to 0.79 and 0.78, in the training and holdout datasets. Based on predicted probabilities from the model, individuals were stratified into 4 clinically meaningful risk strata with a 5‐year event rate of 4.8% vs 47.8% in low vs high risk bins. The protein model AUC, dynamic range, and C‐Index were significantly superior to that of the performance of ApoE genotype and age models for predicting dementia risk.

**Conclusion:**

We successfully developed a blood‐based protein‐only model with a 10‐fold dynamic range that is highly predictive of 5‐year dementia risk in a drug mechanism independent manner. This model out‐performs leading genetic and age risk factors for dementia and given the exclusion of immutable factors, has the potential to monitor changing risk. This tool may facilitate precision prescribing of treatments and enhance clinical trial pipelines by streamlining trial design, efficiency, and costs.

